# Thermodiffusion and thermo-osmosis in thin membranes with slit pores

**DOI:** 10.1140/epje/s10189-026-00592-w

**Published:** 2026-06-01

**Authors:** Bjørn Hafskjold, Signe Kjelstrup

**Affiliations:** PoreLab, Department of Chemistry, Norwegan University of Science and Technology - NTNU, Høgskoleringen 5, 7491 Trondheim, Norway

## Abstract

**Abstract:**

Waste heat sources are potentially useful for component separation in fluid mixtures. To better understand how thermal driving forces can contribute to separation, we have investigated the Soret balances of forces for thermodiffusion and thermo-osmosis. A set of two-component fluid isotope mixtures with mass ratio $m_2/m_1$ has been investigated in membranes with molecular-sized pores. Numerical support generated by molecular dynamics simulations is achieved for two models; one for the Soret coefficient, $S = a_m (m_2-m_1)/(m_2+m_1) + b_{\varepsilon }(\varepsilon _{2\text {m}} -1)$, and one for the thermo-osmotic coefficient, $D_{p} = a (\Delta H/\Delta T) + b$, where $\varepsilon _{2\text {m}}$ is a parameter for the fluid–membrane interactions, $\Delta$ refers to a difference across the membrane, *H* is the bulk fluid enthalpy, and *T* is the temperature. In these formulas $a_m, b_{\varepsilon }, a, b$ are system-specific constants. The results apply to Lennard–Jones/spline isotope mixtures of mass ratios $0.1< (m_2/m_1) < 10$, with thermally insulating membrane materials, and component-specific fluid–pore interactions. The results give information about how the Soret balances depend on membrane properties, which potentially can be used to tailor membranes for efficient separation.

**Graphical abstract:**

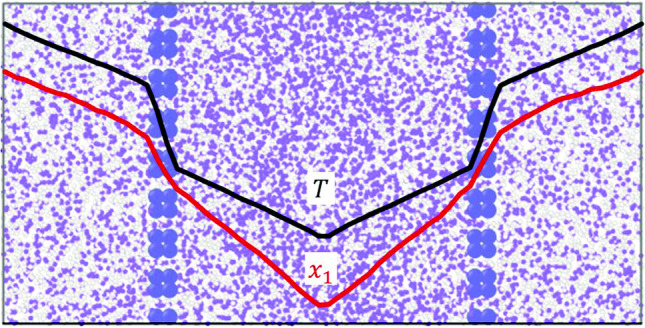

## Introduction

Diffusion and osmosis in membrane systems are present in several important applications, such as water purification [1], waste heat utilization [2], biological membranes [3, 4], and membrane distillation [5]. In *thermo-osmosis*, which can occur in pure systems as well as mixtures, a temperature difference across a membrane is used to generate a mass flow through the membrane. If the system, i.e. the membrane and the bulk fluid on both sides of the membrane, is closed, a pressure difference will build up across the membrane until a balance is reached between the thermal and pressure forces [3, 6, 7]. *Thermodiffusion* occurs in mixtures. This process is also driven by a temperature difference, but now diffusive mass transport will generate a component separation. Thermodiffusion can occur with or without a membrane [8]. If the system is closed, a steady state is reached with balance between the thermal force and the force caused by the difference in composition. The term “Soret equilibrium” has been used to describe this balance [9]. In this work, we consider the composition difference across a membrane as a balancing force in thermodiffusion. We shall prefer to use the term “Soret balance” for this situation, as the term applies also when the chemical force (the chemical potential difference) contains a pressure contribution. Common to thermodiffusion and thermo-osmosis is that an external force (the temperature difference) gives rise to a response from the system until a steady-state balance is reached. In the process, valuable separation work can be done. Both effects can occur in membrane systems where they can be compared on a common basis.

Both processes have been extensively studied experimentally, especially in search of suitable membranes (thermo-osmosis) [1, 10], and for separation studies (thermodiffusion, membrane distillation) [5, 8]. In order to further develop these practical processes, it is helpful to understand the microscopic mechanisms involved and to develop the relevant theory. Early simulation work on thermodiffusion in porous media was done by Colombani et al. [11] and Yeganegi and Pak [12]. A main finding in these studies was that the porous medium reduced the Soret effect. Simulations have proved valuable in this context as they give detailed information about variables and processes under investigation. Thermo-osmosis is less well studied and understood. Notably, Bárragan and Kjelstrup gave an overview of experimental results [10].

More recently, Hannaoui et al. studied thermodiffusion in binary mixtures confined in slit pores of width 3–35 molecular diameters by non-equilibrium molecular dynamics (NEMD) simulations [13, 14]. They found that the pore width had little effect on the Soret coefficient, except for the most narrow pores, which reduced the Soret effect. They also found that increased fluid adsorption on the pore wall reduced thermodiffusion as well as mass diffusion. Their pores were too wide to observe a thermo-osmotic effect. Motivated by the potential of waste heat harvesting and the need for water desalination, Zhao and Wu [15] and Fu et al. [16] used NEMD to study the thermo-osmotic effect of water in carbon nanotubes. They found very large effects that agreed quantitatively with theoretical models based on the pioneering work of Derjaguin et al. [7]. Ganti et al. [17] studied the role of the thermo-osmotic force and the gradient in the stress tensor near the pore wall, again in the framework of the work by Derjaguin et al. They found that there is not a simple relation between the thermo-osmotic force and the (not uniquely defined) microscopic stress tensor. In a recent work, Anzini and coworkers used a combination of linear response theory and conservation laws and gave a good theoretical basis for studies of thermo-osmosis [18, 19]. All these very interesting studies used either a one-component fluid without a Soret effect or wide pores without a thermo-osmotic effect.

The plan for the present work is to investigate the role of the membrane in the Soret balance state, when thermodiffusion and thermo-osmosis occur simultaneously. Hence, we have used a membrane with pore width of molecular scale, $\sim 1$ nm. This is a somewhat different objective than that of the previous studies mentioned above, namely to *avoid* rather than generate fluid flow in the pores. To our knowledge, this has not been done before. An additional objective was to use non-equilibrium thermodynamics (NET) for surfaces [20] as tool for data analysis, hence the membrane used in this study is extremely thin and is approximated as a surface.

The model system has two fluid components and a membrane with slit pores. The fluid compartments adjacent to the membrane are hot and cold. The fluid and the membrane matrix are modeled by a Lennard–Jones/spline pair potential [21]. The potential parameters $\varepsilon$ and $\sigma$ control the interactions between the two fluid components and between the fluid and the membrane.

The Soret equilibrium in a homogeneous phase is a well studied phenomenon and serves here as a useful reference for studies of the effect of porous media. We want to determine the effect of the membrane on the separation efficiency in a binary fluid mixture, and find it useful to compare the results of the porous membrane with those of the homogeneous fluid. To quantify efficiency, we use the difference in composition between the cold and the hot zones in the fluid, which is related to the Soret coefficient [22]. As a similar basis for thermo-osmosis, we use a one-component fluid and a membrane and determine the pressure buildup that results from the temperature difference across the membrane in the closed system.

Finally, we use a two-component fluid isotope mixture with a membrane and study the combined effects of thermo-osmosis and thermodiffusion. Analysis of the effects of the overall driving force (the difference in inverse temperature across the membrane) as they appear in the entropy production of the system is then possible. We use the theory based on NET for surfaces [20] as a tool for analysis of the results from molecular dynamics (MD) simulations. The present study is limited to steady state in a closed system, which means that the mass fluxes are zero. This implies that we do not have data for all the Onsager *L*-coefficients in the NET analysis, but the theory can serve as a basis for future studies with less restrictions on the system, e.g. an open system with zero pressure difference across the membrane, nonzero fluxes of one or both components, different combinations of membrane dimensions and properties, etc. Nevertheless, the results reveal significant effects of fluid-membrane interactions which we use to find simple models for the observed Soret and thermo-osmotic coefficients. In particular, we find that an extension of the Chapman isotope model for thermodiffusion [23, 24] works well also for the membrane system.

The paper outline is as follows. Section 2 includes the theoretical background based on NET. This leads to expressions for the entropy production in the membrane, relations between the fluxes and forces, and definitions for the Soret coefficient and the thermo-osmotic coefficient. Details of the theory are given in Appendix A. In Sect. 3, we describe the MD simulations used to generate data for use with the theory. Details of how the simulations were done and the data analyzed are given in Appendix B. Section 4 gives the results from the simulations of four cases including Soret effects in a bulk isotope mixture, thermo-osmosis in a pure fluid with membrane, isotope mixture with a “neutral” membrane that has no adsorption preference for any of the fluid components, and finally, a more general case with different components and membrane preferences. The results are discussed in Sects. 4.2–4.4. In Sect. 4.5, we develop simple and accurate models for the Soret and thermo-osmotic coefficients for the systems that we have used. Finally, we summarize the important conclusion from this work in Sect. 5.

**Fig. 1:**
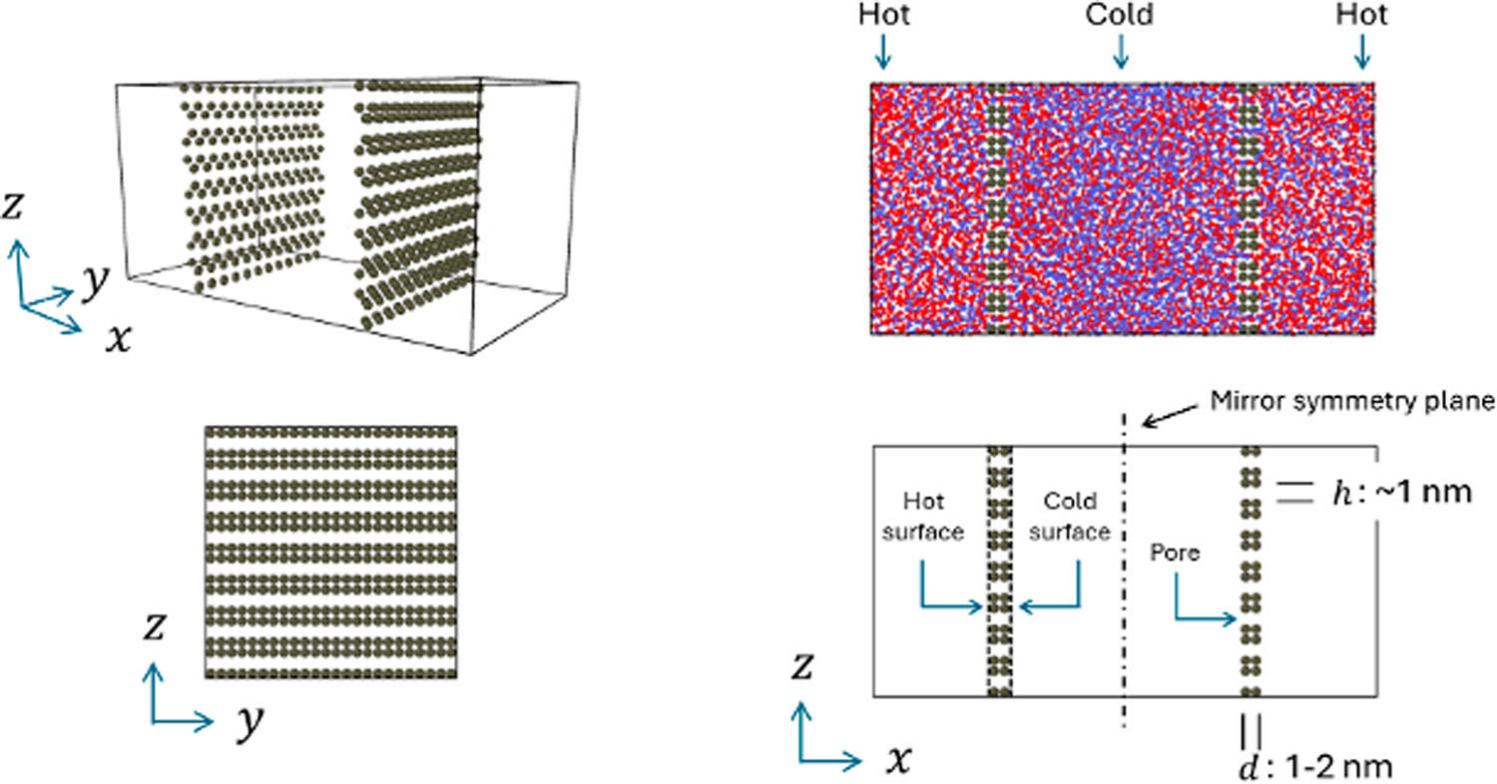
System with two slit-pore membranes. The upper right panel shows the MD box with the fluid, component 1 in red and component 2 in blue. The other panels give a perspective view (upper left) and 2-dimensional projections of the membrane configuration. The membrane thickness and pore width are *d* and *h*, respectively

## Theory

The theory is presented here with the purpose in mind that we want to compare results from experiments and simulations. As a consequence, we use both the measurable heat flux, $J_q^\prime$, and the energy flux, $J_u$. The two fluxes are related by

1 $$\begin{aligned} J_q^\prime = J_u - \sum _k J_k H_k \end{aligned}$$

where $J_k$ and $H_k$ are the mass (molar) flux and partial molar enthalpy, respectively, of component *k*. A closed system in steady state gives $J_k=0$ and $J_q^\prime = J_u$. In open systems, $J_k \ne 0$ in general and the difference between $J_q^\prime$ and $J_u$ is proportional to the enthalpy. The enthalpy is a state variable with different values across the membrane, but with a common reference. The term $J_k H_k$ represents the amount of heat transported with the mass, which is different from the heat conducted as $J_q^\prime$. The distinction between $J_q^\prime$ and $J_u$ is important in understanding the mechanisms for energy transport across surfaces and membranes. Furthermore, we have chosen to introduce the heat flux of the membrane, $J_q^{\prime \,\text {m}}$ (see Eqs. (A5) and (5)), since our focus is on membrane properties. The chemical driving force is split into contributions from each component in a manner that provides conjugate fluxes and forces that we are seeking, cf. Equations (5)–(7). Mathematical details in the derivations are given in Appendix A because these are often unfamiliar.

### The entropy production and the flux equations

We consider a closed system with two completely miscible fluid components and two membranes as illustrated in Fig. 1. Temperature gradients are established by heating each end of the box and cooling the center. This divides the system into two symmetric halves with a membrane in each of them. Each of the two halves has a hot and a cold bulk fluid with a membrane in between. The membrane is saturated with fluid. The thin membrane can be considered as an autonomous system, an interface or surface, with the excess entropy production in the membrane, $\sigma ^\text {m}$, [20], p. 84, Eq. (5.17):

2 $$\begin{aligned} \sigma ^\text {m}= &   J_u^\text {h} \Delta _\text {h,m} \left( \frac{1}{T} \right) + J_u^\text {c} \Delta _\text {m,c} \left( \frac{1}{T} \right) - \sum _{k=1}^2 J_k^\text {h} \Delta _\text {h,m} \left( \frac{\mu _k}{T} \right) \nonumber \\  &   \quad - \sum _{k=1}^2 J_k^\text {c} \Delta _\text {m,c} \left( \frac{\mu _k}{T} \right) \end{aligned}$$

where *T* is the temperature and $\mu _k$ is the chemical potential of component *k*. The symbol $\Delta _\text {h,m}$ represents the difference “state m” - “state h.” We see from Eq. (2) that the thermal driving force is $\Delta _\text {h,m} \left( \frac{1}{T} \right)$, rather than $\Delta _\text {h,m} T$. Components 1 and 2 are the fluid components. The porous matrix is the third component and in Eq. (2), we have chosen it as a stationary frame of reference, i.e. $J_3 \equiv 0$, and therefore not included it explicitly in the entropy production.

In steady state, $J_u^\text {h}=J_u^\text {c}=J_u$ and $J_k^\text {h}=J_k^\text {c}=J_k$ due to energy and mass conservation, and Eq. (2) becomes

3 $$\begin{aligned} \sigma ^\text {m} = J_u \Delta _\text {h,c} \left( \frac{1}{T} \right) - \sum _{k=1}^2 J_k \Delta _\text {h,c} \left( \frac{\mu _k}{T} \right) \end{aligned}$$

where $\Delta _\text {h,c}$ means the difference of a property at the cold minus the hot side of the outer surface of the membrane. Such surface properties are determined by extrapolation of the adjacent bulk properties, see Appendix B.2. The chemical potential $\mu _k$ depends on temperature, pressure, and composition. To find working equations for thermo-osmosis and thermodiffusion, we expand $\mu _k/T$ as a function of temperature, pressure, and composition for an actual state around a reference state. Details of this derivation are given in Appendix A, leading to the following approximate expression for the entropy production in the membrane:

4 $$\begin{aligned}  &   \sigma ^\text {m} \approx \overline{J_q^\prime }\, \Delta _\text {h,c} \left( \frac{1}{T} \right) - \left[ \;\overline{\left( \frac{V_1}{T} \right) } J_1 + \overline{\left( \frac{V_2}{T} \right) }J_2\right] \Delta _\text {h,c} p \nonumber \\  &   \quad - R \left[ J_1 \overline{\left( \frac{1}{x_1} \right) } - J_2 \overline{\left( \frac{1}{x_2} \right) } \right] \Delta _\text {h,c} x_1 \end{aligned}$$

where $V_k$ and $x_k$ are the partial molar volume and mole fraction, respectively, of component *k*, *p* is the pressure, and *R* is the gas constant. The overline represents average membrane property as given in detail in Appendix A. On this basis, the corresponding flux-force relations are

5 $$\begin{aligned} \overline{J_q^\prime }&= L_{qq}\Delta _\text {h,c} \left( \frac{1}{T} \right) - L_{qV} \Delta _\text {h,c} p - L_{qD} \Delta _\text {h,c} x_1 \end{aligned}$$

6 $$\begin{aligned} J_V&= L_{Vq}\Delta _\text {h,c} \left( \frac{1}{T} \right) - L_{VV} \Delta _\text {h,c} p - L_{VD} \Delta _\text {h,c}x_1 \end{aligned}$$

7 $$\begin{aligned} J_D&= L_{Dq}\Delta _\text {h,c} \left( \frac{1}{T} \right) - L_{DV} \Delta _\text {h,c} p - L_{DD} \Delta _\text {h,c} x_1 \end{aligned}$$

where

8 $$\begin{aligned} \overline{J_q^\prime } = (J_q^{\prime \, \text {h}}+J_q^{\prime \, \text {c}})/2 \end{aligned}$$

is the measurable heat flux,

9 $$\begin{aligned} J_V = \overline{ \left( \frac{V_1}{T} \right) }J_1 + \overline{ \left( \frac{V_2}{T} \right) } J_2 \end{aligned}$$

is the volume flux and

10 $$\begin{aligned} J_D = R \left[ J_1 \overline{\left( \frac{1}{x_1} \right) } - J_2 \overline{\left( \frac{1}{x_2} \right) } \right] \end{aligned}$$

is the diffusive mass flux.

The transport coefficients that we derive in Sect. 2.2, will refer to a discrete description of $\sigma ^\text {m}$ with the membrane appearing as a black box. In the derivation, we have used entropy production invariance. The slit width and the membrane thickness are of molecular dimensions.

### The transport coefficients

We are interested in thermodiffusion and thermo-osmosis, the diffusive mass flux and volume flux, respectively, caused by a temperature difference. Key properties to describe the phenomena are $S=\Delta _\text {h,c} x_1/\Delta _\text {h,c} T$ and $D_p=\Delta _\text {h,c} p/\Delta _\text {h,c} T$, respectively. The *S* is equivalent to the Soret coefficient in bulk fluids. These effects can be determined separately by varying the fluxes and/or the forces. For instance, if $J_D = 0$ and $\Delta _\text {h,c} p = 0$, *S* may be obtained from Eq. (7) as

11 $$\begin{aligned} S = \left( \frac{\Delta _\text {h,c} x_1}{\Delta _\text {h,c} T}\right) _{J_D=\Delta _\text {h,c} p = 0} = -\left( \frac{L_{Dq}L_{DD}}{T^\text {c}T^\text {h}} \right) _{J_D=\Delta _\text {h,c} p = 0} \end{aligned}$$

where $T^\text {c}$ and $T^\text {h}$ are the temperatures at the cold and hot side, respectively, of the membrane. There are six independent *L*-coefficients which can be determined with six combinations of the forces. The conditions we shall use in this work are convenient from a computational point of view, viz. $J_V = J_D = 0$ (closed system). We solve Eq. (6) for $\Delta _\text {h,c} p$ and use the result in Eq. (7) to obtain

12 $$\begin{aligned} S&= \left( \frac{\Delta _\text {h,c} x_1}{\Delta _\text {h,c} T}\right) _{J_V=J_D=0} \end{aligned}$$

13 $$\begin{aligned}&= -\frac{1}{ T^\text {c}T^\text {h}} \left( \frac{L_{Dq}L_{VV}-L_{DV}L_{Vq}}{L_{DD}L_{VV}-L_{DV}L_{VD}} \right) _{J_V=J_D=0}\;. \end{aligned}$$

Likewise, we solve Eq. (7) for $\Delta _\text {h,c}x_1$ and use the result in Eq. (6) to get the thermo-osmotic coefficient

14 $$\begin{aligned} D_p&= \left( \frac{\Delta _\text {h,c} p}{\Delta _\text {h,c} T}\right) _{J_V=J_D=0} \end{aligned}$$

15 $$\begin{aligned}&= -\frac{1}{ T^\text {c}T^\text {h}} \left( \frac{L_{Vq}L_{DD}-L_{VD}L_{Dq}}{L_{DD}L_{VV}-L_{DV}L_{VD}} \right) _{J_V=J_D=0}\;. \end{aligned}$$

In the discussion of our simulation results, we shall use Eqs. (12) and (14) as the operational definitions of *S* and $D_p$. We note that there are common factors in both variables, the product of the two temperatures and the denominator in Eqs. (13) and (15). This denominator is always positive and can be regarded as one term. The *S* and $D_p$ arise from coupling between the volume flux, $J_V$, the interdiffusion flux, $J_D$, and the heat flux, $J_q^\prime$, via the coefficients $L_{Vq}$, $L_{Dq}$, and $L_{VD}$. The expressions for *S* and $D_p$ have both two contributions in the numerator. We shall see that empirical formulas can be found for each coefficient and that both contain two contributions.

## Simulations

Molecular dynamics simulations were performed with a Lennard–Jones/spline (LJ/s) model [21] in a box with periodic boundary conditions in all three directions and aspect ratio $L_x{:}L_y{:}L_z = 2{:}1{:}1$ as shown in Fig. 1. The box had two slit-pore membranes and two fluid components with a total number of particles $N=13,824$. The box was organized in 64 layers of equal thickness normal to the *x*-direction for recording temperature, density, composition, and other data in the simulation. One layer at each end of the box (the hot zone) was thermostated to a high temperature $T_\text {H}$ and two layers in the center (the cold zones) to a low temperature $T_\text {C}$. The thermostats were simple velocity scaling with momentum preservation [25]. This created the thermodynamic driving force in the system, the gradient in inverse temperature.

**Fig. 2:**
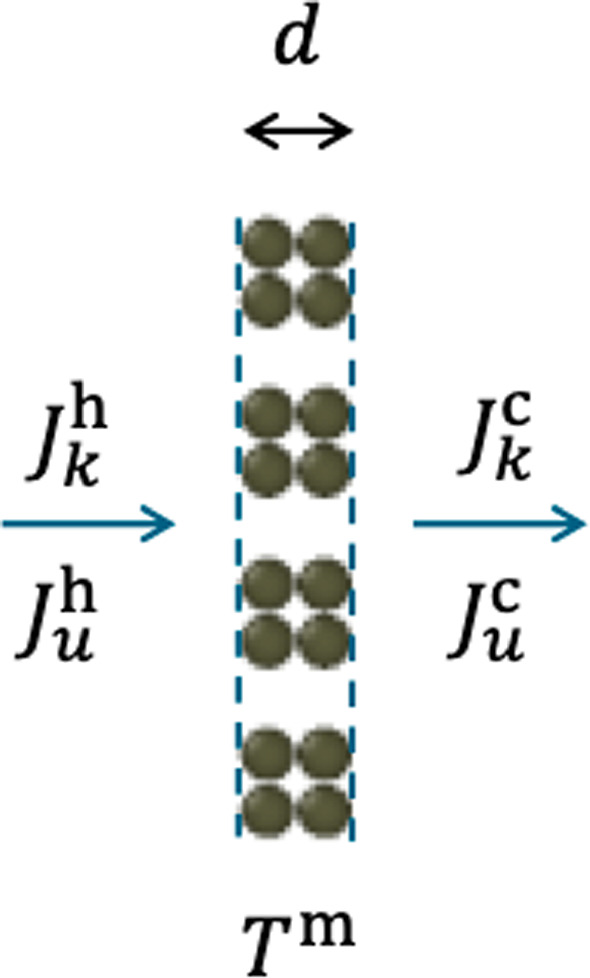
Section of the membrane showing molar fluxes of component *k*, $J_k^\text {h}$ and $J_k^\text {c}$ at the hot and cold sides of the membrane, similarly for the energy fluxes, $J_u^\text {h}$ and $J_u^\text {c}$, and the membrane temperature $T^\text {m}$

Each membrane had eight slit pores as illustrated in Figs. 1 and 2. The particles making up the membrane were fixed to their FCC lattice positions. This means that the membrane material was a perfect thermal insulator. All conduction of heat therefore took place in the pores only. The membrane thickness was fixed, $d=1.79 \sigma$, i.e. 1.79 in Lennard–Jones units, which will be used from here on. The width of each slit was $h=1.79$. The thermostat set points were $T_\text {H} = 4.0$ and $T_\text {C} = 2.0$ in the thermostated layers. This temperature difference creates extremely large gradients in the system and we examined the effects, see Appendix B.3. The overall density was $\rho = 0.7$, which means that the fluid was a dense supercritical fluid. With this density, the length of the MD box was $L_x/\sigma = \root 3 \of {\frac{N L_x^2}{\rho L_y L_z}} \approx 42.9$. Using argon as a corresponding real fluid with $\sigma \approx 0.34$ nm, the box length was approximately 15 nm, slit width $h \approx 0.6$ nm and the membrane thickness $d \approx 0.6$ nm.

The role of the interactions between the fluid and the membrane was investigated by systematically varying the potential parameter $0.5 \le \varepsilon _{2\text {m}} \le 2.0$, where subscript “2 m” represents fluid component 2 and membrane component “m.” The mass ratio between the two fluid components was also varied, $0.1 \le m_2/m_1 \le 10.0$. All the other potential parameters were kept constant at value 1.0. A summary of the parameters used in the simulations are listed in Table 1.

The system can be understood as follows: The mixture of components 1 and 2 can be regarded as an ideal mixture in the bulk phases away from the membrane. However, the components interact differently with the porous membrane walls, meaning that there is a preference for one component over the other when it comes to adsorption. Physically, one can imagine the two fluid components as being similar enough to behave as an ideal mixture in bulk, but one of the components has an active group that interacts with the surface of the membrane. By this, the membrane is made selective to one of the components. This selectivity has an impact on the Soret balance as we shall see.

Table 2 describes the increasing complexity of the four cases we have studied. In order to establish a reference for discussion, we repeated earlier work on thermodiffusion without membranes such that the conditions matched the present study. This reference is Case 1. In Case 2, we investigated pure thermo-osmosis with a one-component fluid and membranes. This provided our reference for thermo-osmosis data. Case 3 addressed transport of a fluid mixture across a neutral membrane, while Case 4 addressed the same mixture for a variety of isotope combinations as well as fluid-membrane interactions.

**Table 1:** Summary of model parameters used in this study

Number of particles:	13,824
Membrane thickness:	$d=1.79$
Pore width:	$h= 1.79$
Number of pores in each membrane:	8
Molecular diameter:	All particles of same size ($\sigma _{ij}=1.0$)
Molecular interactions:	$\varepsilon _{ij}=\varepsilon _{1\text {m}}=1.0$
	$$\varepsilon _{2\text {m}}=0.5, 0.75, 1.0, 1.25, 1.5, 1.75, 2.0$$
Mass ratios:	$m_2/m_1=0.1, 0.25, 0.5, 1.0, 2.0, 4.0, 10.0$
Overall fluid density:	0.7
Overall fluid composition:	$x_1=x_2=0.5$
Temperature (hot):	4.0
Temperature (cold):	2.0

The symbols $\sigma _{ij}$ and $\epsilon _{ij}$ represent parameters in the Lennard–Jones potential. All numbers are without units or in dimensionless Lennard–Jones units

**Table 2:** Specifications of simulated cases

Case 1	Thermodiffusion case. $m_2/m_1 = 10.0$, two fluid components, no membrane
Case 2	Thermo-osmosis case. $\varepsilon _\text {fluid-membrane} = 1.0$, one fluid component and membrane
Case 3	$\varepsilon _{1\text {m}} = \varepsilon _{2\text {m}} = 1.0$ (neutral membrane), $m_2/m_1 = 10.0$,
	two fluid components and membrane
Case 4	$\varepsilon _{1\text {m}} = 1.0$, different values of $\varepsilon _{2\text {m}}$ and $m_2/m_1$,
	two fluid components and membrane

## Results and discussion

### Cases

#### Case 1. Soret effect in an isotope mixture. $\Delta p = 0$. No membrane

**Fig. 3:**
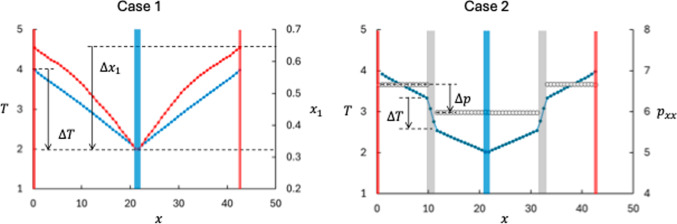
Profiles of temperature (blue symbols), mole fraction of component 1 (red symbols), and pressure (white symbols) as function of position in the MD box. The left and right panels show results for cases 1 and 2, respectively. Case 1 is bulk fluid (no membrane) for a two-component mixture with $m_2/m_1=10$. Case 2 has two neutral membranes ($\varepsilon _{\text {membrane-fluid}}=1.0$, marked as gray shaded areas) and a pure fluid. The red and blue shaded areas mark the thermostated hot and cold regions. The dashed horizontal lines indicate the extrapolated values of *T*, $x_1$, and $p_{xx}$ used to determine $\Delta T$, $\Delta x_1$, and $\Delta p.$

It is well known that the mass ratio between two components in a bulk fluid is an important parameter for the Soret effect [8, 23]. We therefore start the results section by recollecting results for a mixture of two fluid components in bulk at Soret equilibrium, i.e. two LJ/s components with equal potential parameters and different masses. The so-called isotope Soret effect, which occurs in this system, can be a significant contribution to the total Soret effect [8, 26–30]. The isotope Soret effect in the LJ/s system was first studied by Kincaid and Hafskjold [31]. We include here results from a simulation with mass ratio $m_2/m_1 = 10.0$, see Fig. 3, left panel.^1^

The data show the normal isotope effect, *viz.* the lighter component (component 1) is enriched at the hot side of the mixture. The Soret coefficient was determined to $S=0.16$ in this case. For the sake of comparison with the cases 3 and 4, we have used *S* as defined by Eq. (12) also for the bulk case, which is missing a factor $1/(x_1 x_2)$ compared with the usual definition [8].

**Fig. 4:**
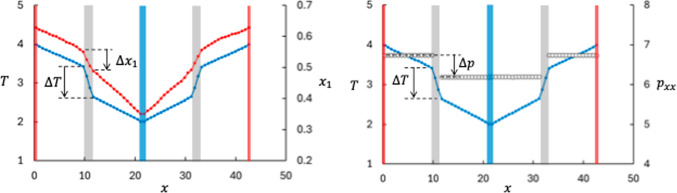
Profiles of temperature (blue symbols), mole fraction of component 1 (red symbols, left panel), and pressure (white symbols, right panel) as function of position for Case 3. This case has the same membrane configuration as Case 2. The fluid mixture has $m_2/m_1=10$ and the membrane is neutral, $\varepsilon _{2\text {m}}=1.0$. Membrane thickness is $d=1.79$

#### Case 2. Thermo-osmotic effect. Pure fluid with membrane. $\Delta _\text {h,c} p \ne 0$

As mentioned in the Introduction, the thermo-osmotic effect can occur both in pure fluids and in mixtures. We have chosen a one-component fluid with a membrane as basis for comparison with results for mixtures. Figure 3, right panel, shows the temperature and pressure profiles in this case. There is a significant pressure difference across the membrane, driven by the temperature difference. This is the thermo-osmotic effect. The value of $D_p$ determined with Eq. (14) is 0.85 for this case. The pressure appears to be constant throughout the bulk phases. The $\Delta _\text {h,c} p$-term in Eq. (A5) (which corresponds to a $\nabla p$-term in bulk fluids) is usually ignored in bulk fluids^2^.

#### Case 3. Isotope mixture in neutral membrane. $\varepsilon _{1\text {m}}=\varepsilon _{2\text {m}}=1.0$, $\Delta _\text {h,c} p \ne 0$

**Fig. 5:**
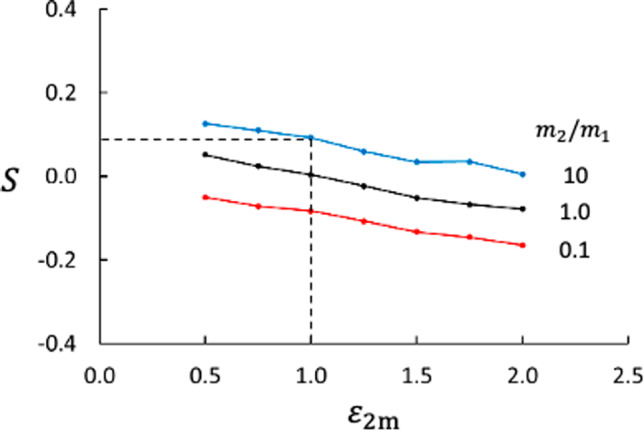
Soret coefficient as function of interaction strength between component 2 and the membrane for different mass ratios. The dashed lines point at the results from Case 3

We then generalized cases 1 and 2 to a two-component fluid isotope mixture with mass ratio $m_2/m_1 = 10.0$ and $\varepsilon _{2\text {m}} = \varepsilon _{1\text {m}} = 1.0$ (neutral membrane). This membrane has no preference for any of the two fluid components. The results are shown in Fig. 4. The values for $\Delta T$ and $\Delta x_1$ were determined from linear extrapolations of the respective bulk values to the membrane surfaces. Again, the figure shows the normal isotope behavior with respect to the sign of the Soret coefficient. Due to the confinement of the pores and the fact that the membrane is a thermal insulator, there is a relatively large temperature drop across the membrane and consequently a large mole fraction difference. However, the membrane reduces the Soret coefficient, the temperature and mole fraction differences across the membrane gave $S = 0.09$, which is about half of the bulk value. A reduction in the Soret coefficient means that some of the thermal energy is spent on building a pressure difference. The result for Case 3 is indicated by the dashed lines in Fig. 5.

The thermo-osmotic coefficient turned out to depend weakly on the mass ratio, $D_p = 0.70$, which is about 20 % lower than for the one-component Case 2. The fact that $D_p$ depends on the mass ratio at all shows that thermo-osmosis is a non-equilibrium phenomenon, like regular osmosis.

#### Case 4. Combinations of mass ratios and fluid-membrane interaction (specific membranes)

**Fig. 6:**
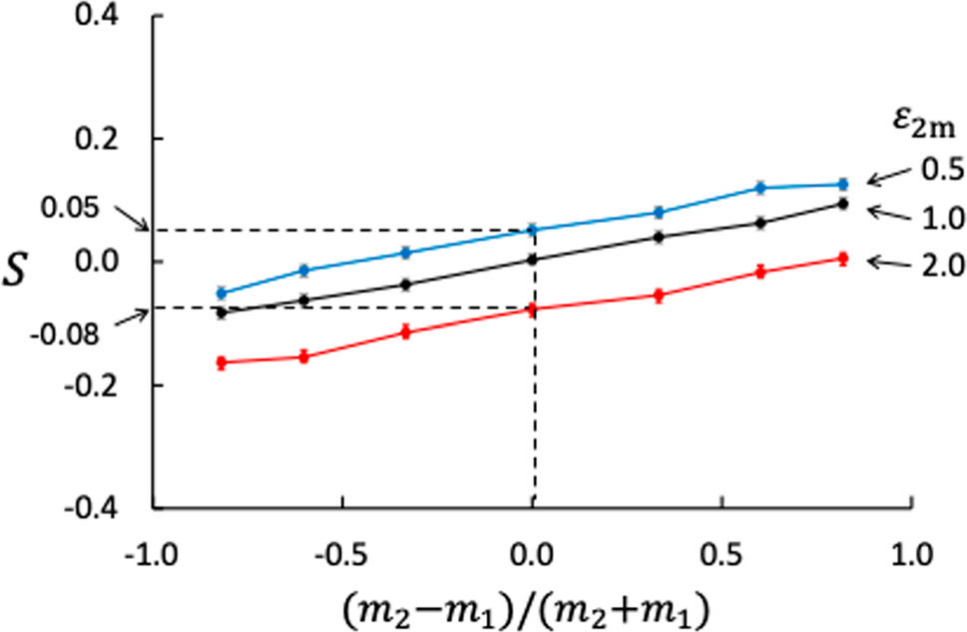
Soret coefficient as function of mass ratio for different values of $\varepsilon _{2\text {m}}$. The dashed lines point at data for $m_2/m_1 =1.0$, please see text

**Fig. 7:**
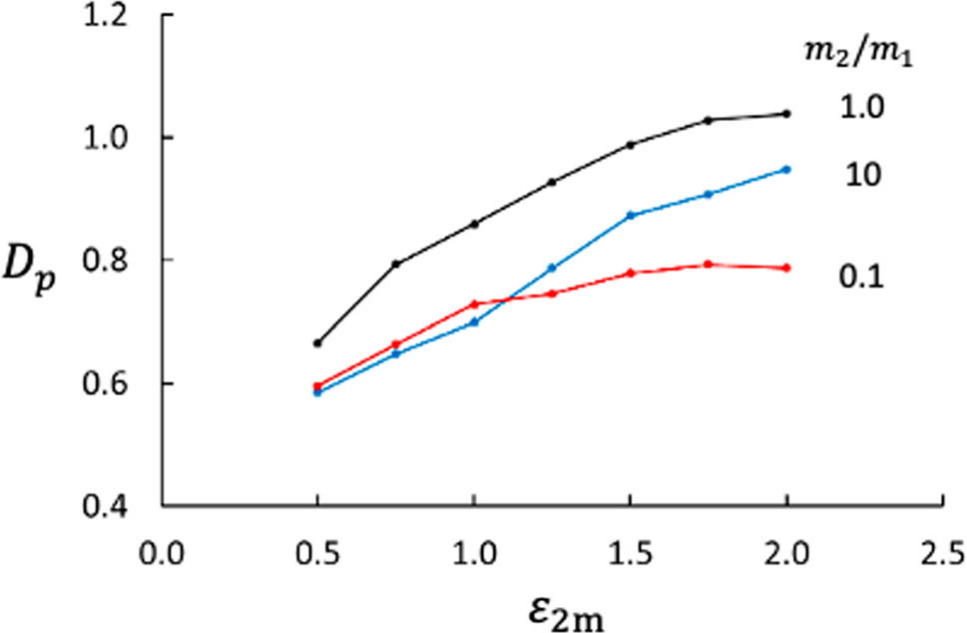
Thermo-osmotic coefficient as function of interaction strength between component 2 and the membrane for different mass ratios

In this case, we show how the Soret and thermo-osmotic coefficients depend on wider spectra of mass ratios and fluid–membrane interactions. Results from NEMD runs with mass ratio $m_2/m_1 =0.10, 0.25, 0.50, 1.00, 2.00, 4.00, 10.00$, fluid–membrane interactions $\varepsilon _{1\text {m}} = 1.00$ and $\varepsilon _{2\text {m}} = 0.50, 0.75, 1.00, 1.25, 1.50, 1.75,2.0$, and with membrane thickness $d=1.79$, in total 49 cases (including Case 3), were generated. Qualitatively, the profiles of temperature, mole fraction, and pressure were more or less the same as shown in Figs. 3 and 4. Results for the Soret coefficient are shown in Figs. 5 and 6 and for the thermo-osmotic coefficient in Figs. 7 and 9.

### Soret effect

Increased attraction between the membrane and component 2 decreases the Soret coefficient, *S* depends linearly on $\varepsilon _{2\text {m}}$ for all mass ratios. We emphasize here that the bulk phases also contribute to the overall Soret effect, but these contributions are not considered here because the bulk-phase conditions did not vary much from case to case. The average temperature at the hot side of the membrane was $3.362 \pm 0.006$ based on all the 49 cases. The corresponding value at the cold side of the membrane was $2.588 \pm 0.007$, giving an average $\Delta _\text {h,c} T = -0.774 \pm 0.005$ over the membrane.

An interesting question is whether the membrane can cause separation if the two fluid components are identical, i.e. having the same mass in addition to identical potential parameters ($m_2/m_1 = 1$ and $\sigma _{ij} = \varepsilon _{ij} = 1$ for all fluid pairs *ij*). We tested this, again with different values of $\varepsilon _{2\text {m}}$ ($\varepsilon _{2\text {m}}=1$ is the neutral case). The results are shown as the black line in Fig. 5 and guided with the dashed lines in Fig. 6. The two extreme cases with $\varepsilon _{2\text {m}}=0.5$ and 2.0 give $S=0.05$ and −0.08, respectively. The change in sign of *S* is not surprising because the two cases are symmetric in the sense that for $\varepsilon _{2\text {m}}=0.5$, component 1 interacts more strongly with the membrane and for $\varepsilon _{2\text {m}}=2.0$, component 2 interacts more strongly, all other properties being equal. This suggests that nanoporous membranes can be used to separate mixtures of components with very similar molecular properties, provided the two components interact sufficiently different with the membrane.

**Fig. 8:**
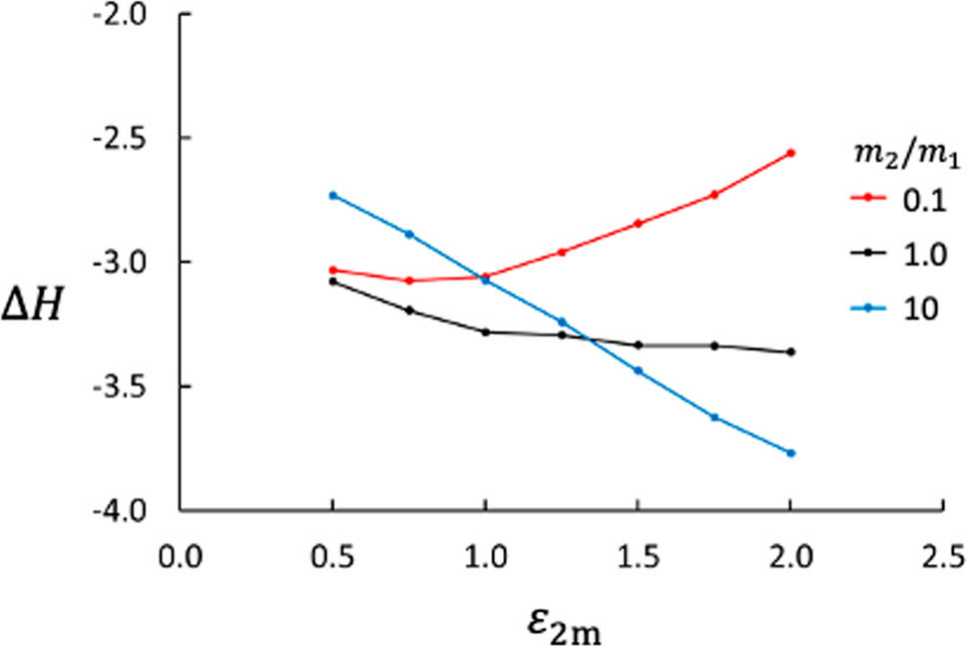
Enthalpy change across the membrane. A negative $\Delta H$ means that the enthalpy is lower at the cold side of the membrane

**Fig. 9:**
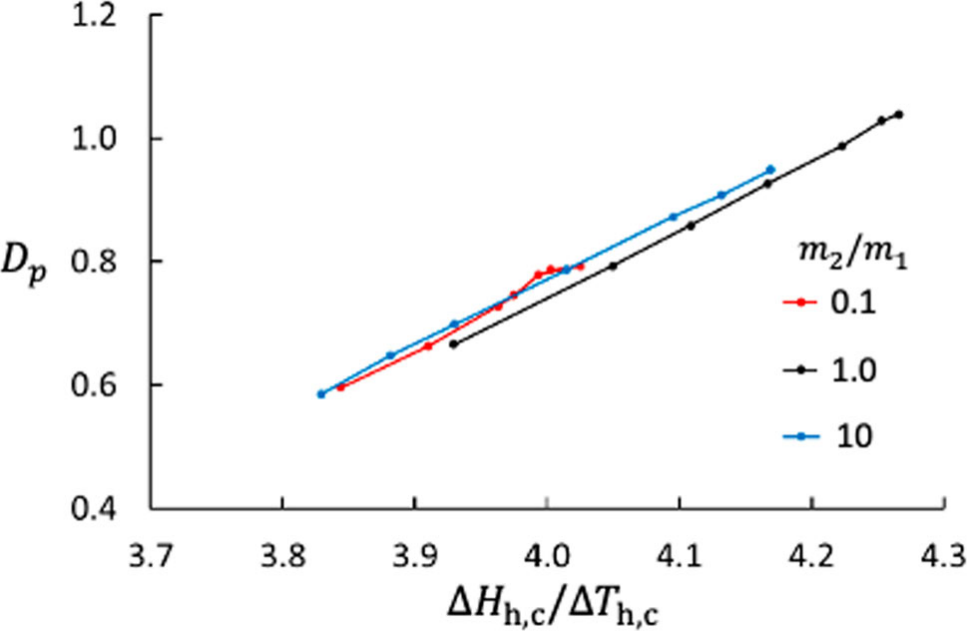
Thermo-osmotic coefficient as function of the ratio between the enthalpy difference and temperature difference across the membrane, $\Delta H_\text {h,c} / \Delta T_\text {h,c}$, for three different mass ratios

### Thermo-osmotic effect

The nanoporous membrane will give enough resistance to fluid flow to sustain a pressure difference across the membrane at steady state. This effect can be quantified by the thermo-osmotic coefficient defined by Eq. (14). Examples of temperature and pressure profiles are shown in Figs. 3 and 4. The pressure component $p_{xx}$ is independent of position in the bulk phases and the difference across the membrane is $\Delta _\text {h,c} p$. The pressure is not known in the pores due to the lack of a consistent way to compute the microscopic pressure tensor [33], which is why data are not shown for *x*-values in the pore. The *yy*- and *zz*-components of the pressure tensor (not shown) oscillate as function of *x* near the membrane surface, which is normal behavior near surfaces. The extrapolated pressure values from the homogeneous bulk to the membrane surface was therefore determined from the *xx*-component.

The membrane used here has very low permeability with a significant thermo-osmotic effect. Figure 7 shows $D_p$ as function of the interaction strength between the membrane and component 2 for three different mass ratios. We see that the interaction strength is important, which is expected based on relation between the heat flux and the coefficients $L_{qV},L_{qD}$ cf. Equation (5). The $D_p$ increases with increasing $\varepsilon _{2\text {m}}$, suggesting that the interaction between the fluid and the membrane is the driving force for thermo-osmosis. The interaction strength is also seen in the difference in enthalpy across the membrane, Fig. 8. Figure 9 shows that $D_p$ depends essentially linearly on the enthalpy difference scaled with the temperature difference, $\Delta _\text {h,c} H / \Delta _\text {h,c} T$, independent of the mass ratio between the two components.

The thermo-osmotic effect depends on the mass ratio and the membrane-fluid interaction, like the Soret effect does, whereas *S* decreases with increasing $\varepsilon _{2\text {m}}$, $D_p$ increases. One might think that the pressure difference $\Delta _\text {h,c} p$ is a consequence of the equilibrium equation of state and caused by the temperature difference between the bulk phases. If it were, $\Delta _\text {h,c} p$ would be independent of the particles’ membrane properties, which Fig. 7 shows is not the case. Moreover, it is evident from the Soret balance that the pressure difference is not an equilibrium situation.

**Fig. 10:**
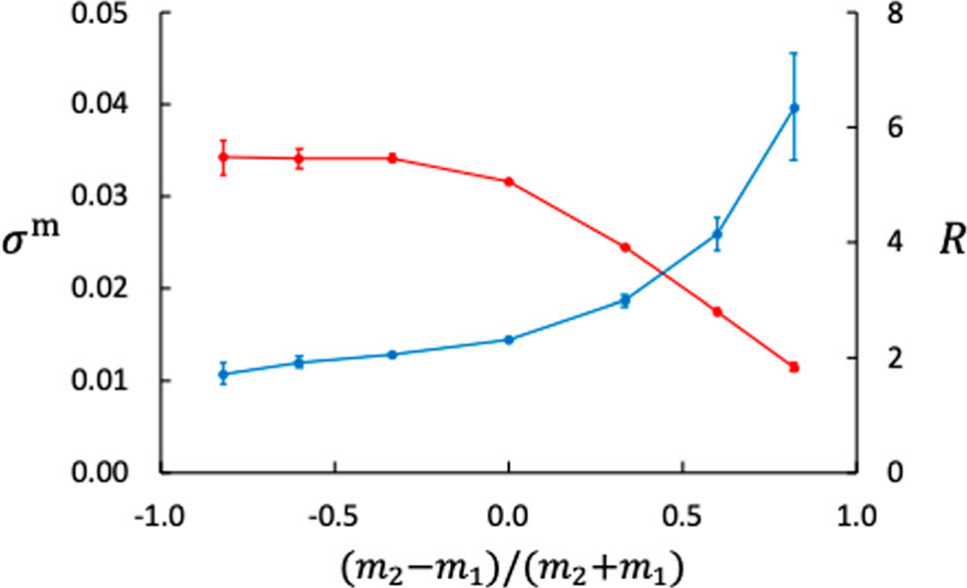
Entropy production (red) and thermal resistance (blue) across the membrane as function of mass ratio. The data are averages of results for all values of $\varepsilon _{2\text {m}}$ studied here

### Entropy production and thermal resistance

The entropy production under a Soret balance is given by Eq. (4), which in the present case becomes

16 $$\begin{aligned} \sigma ^\text {m} = J_q^\prime \Delta _\text {h,c} \left( \frac{1}{T} \right) = - J_q^\prime \frac{\Delta _\text {h,c}T }{T^\text {c}T^\text {h}} \end{aligned}$$

We found (within uncertainties) that $\sigma ^\text {m}$ is independent of $\varepsilon _{2\text {m}}$. The data shown in Fig. 10 are averages over all $\varepsilon _{2\text {m}}$-values with variance shown by error bars as standard error of the mean. The figure also shows the thermal resistance to heat flow through the membrane. The resistance *R* is given by

17 $$\begin{aligned} J_q^\prime = - \frac{1}{R} \Delta _\text {h,c}T \end{aligned}$$

The larger the isotope ratio, the larger is the entropy production and the lost work (dissipated energy) for a given temperature difference. The independence of the fluid wall interaction may be explained by the steady state (zero mass fluxes).

These results apply to the restrictions $J_V = J_D = 0$ and that the membrane material is a prefect thermal insulator. Given that $\Delta _\text {h,c}T$, $T^\text {c}$, and $T^\text {h}$ are approximately the same in all the 49 cases means that the variation in entropy production between the different cases is determined by the variation in the heat flux, or equivalently, the variation in resistance. The resistance is almost linear in $m_2/m_1$ (not shown).

### Linear models for *S* and $D_p$

In order to obtain a bird’s eye view on the processes, consider first Eq. (1). When applied to the hot and cold membrane surfaces, we obtain from energy conservation

18 $$\begin{aligned} \Delta _\text {h,c}J_q^{\prime } = J_q^{\prime ,\text {c}} - J_q^{\prime ,\text {h}} = \sum _k J_k (H_k^\text {h} - H_k^\text {c}) \end{aligned}$$

at steady state. The enthalpy difference $H_k^\text {h} - H_k^\text {c}$ is positive, so a component with $J_k > 0$ contributes positively to $\Delta _\text {h,c}J_q^{\prime }$. This means that the heat flux coming out of the membrane on the cold side is larger than the heat flux going into the membrane on the hot side. In other words, net heat will be (reversibly) transferred by the movement of the fluid down the temperature gradient.

Our results confirm that the isotope Soret effect is significant in the membrane, like in bulk fluids, i.e. the membrane has also an ability to do work, and concentrate the heavy particle on the low-temperature side. The components migrate to the region where they most effectively contribute to heat transport, which is the hot region for the lighter components and the cold region for the heavier [34]. The sign of the Soret coefficient is determined by this work. In bulk fluids, the isotope Soret effect for molecules without moments of inertia is well represented by the model [8, 23, 24, 27–30]

19 $$\begin{aligned} S_\text {isotope} = a_m \frac{m_2-m_1}{m_2+m_1} \end{aligned}$$

where $a_m$ is an empirical coefficient when the model is used for high-density fluid mixtures. The sign of *S* is in this model given by the numerator and the ratio $m_2/m_1$. The effect of the mass ratio is seen in Figs. 5 and 6 for $\varepsilon _{2\text {m}}=1.0$ (neutral membrane) where $m_2=m_1$ is a symmetry point for *S*. The Soret coefficient decreases with decreasing mass ratio, reaching the limit $\lim _{m_2/m_1 \rightarrow 0} S = - a_m$. The positive slope of *S* shown in Fig. 6 means that $a_m>0$.

**Fig. 11:**
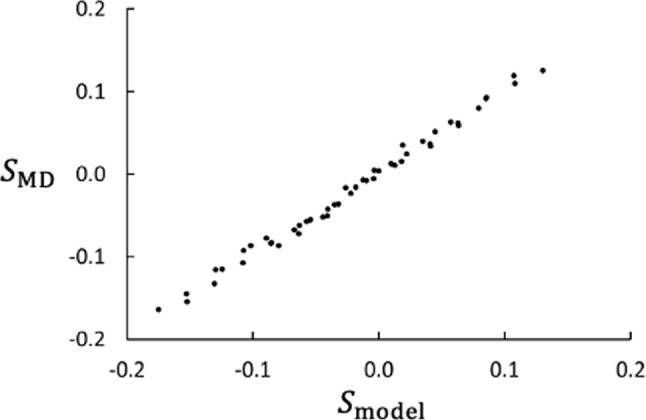
Checking the model proposition for *S*. Plot of NEMD results vs. model results. The model is given by Eq. (20). The data represent the 49 combinations of $m_2/m_1$ and $\varepsilon _{2\text {m}}$ used in this study

The membrane-fluid interaction will modify *S* like the additional “chemical contribution” in bulk fluids [8]. Inspired by the work of Galliero et al. [28], we find that a simple model with superposition of contributions

20 $$\begin{aligned} S_\text {model} = a_m \frac{m_2-m_1}{m_2+m_1} + b_\varepsilon (\varepsilon _{2\text {m}}-1) \end{aligned}$$

with $a_m=0.105$ and $b_\varepsilon =-0.085$, is an excellent representation of the 49 cases studied here. The model gives the cross plot shown in Fig. 11.

The second term at the rhs of Eq. (20) means that the membrane gives an additional degree of freedom that can be used to separate components that are otherwise difficult to separate.

It is known from irreversible thermodynamics that the Soret coefficient is closely related to the heat of transfer. The heat of transfer is defined through Onsager symmetry as the heat transported with the mass flux at zero temperature gradient. In the present case, there are two mass fluxes and two conjugate heats of transfer, $q_V^*$ and $q_D^*$, and the measurable heat flux can be expresses by

21 $$\begin{aligned} J_q^\prime =L_{qq} \Delta _\text {h,c} \left( \frac{1}{T} \right) + q_D^*J_D + q_V^* J_V \;. \end{aligned}$$

**Fig. 12:**
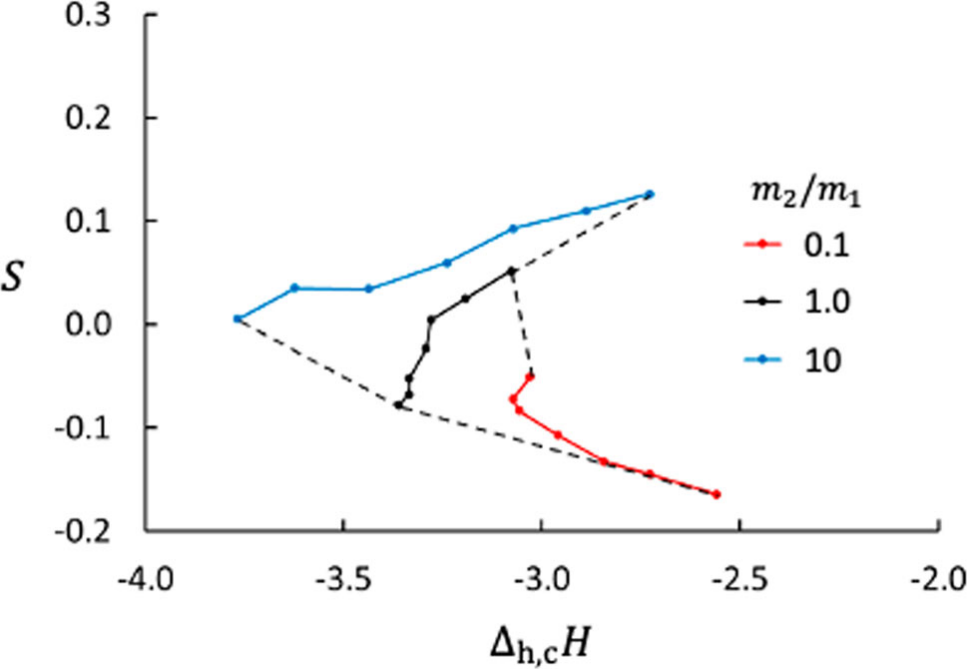
Soret coefficient vs. enthalpy difference across membrane. Each solid line represents a series with constant value of $m_2/m_1$ (as shown in the legend). Each line is also a function of $\varepsilon _{2\text {m}}$ through $\Delta H$. The dashed lines connect data points with the same value of $\varepsilon _{2\text {m}}$

To determine both $q_D^*$ and $q_V^*$, we would need to control $J_D$ and $J_V$ separately, which is not possible with the closed system we have used in this work. Determination of $q_V^*$ and $q_D^*$ and establishing the relation between the heats of transfer and $\Delta _\text {h,c}H$ are interesting tasks for a continuation of the present work. We may, however, point at how the Soret coefficient correlates with $\Delta _\text {h,c}H$ as shown in Fig. 12. We see that *S* depends strongly on $\Delta _\text {h,c}H$ and we think that a better understanding of this relationship would give more insight in the mechanisms behind the Soret effect.

Barragán and Kjelstrup have discussed the magnitude and sign of the thermo-osmotic coefficient based on experimental data [10]. They observed that mass flows from the cold to the hot side of hydrophilic membranes in aqueous solutions and that the coefficient can change sign depending on the nature of the membrane. Although they define the thermo-osmotic coefficient somewhat differently from the definition we have used here, the underlying physical concept is the same, namely representing a volumetric mass flux driven by a temperature gradient. In the thermo-osmotic balance at zero mass flux, this manifests itself as a pressure difference. If we consider an increasing value of $\varepsilon _{2\text {m}}$ as representing a more “hydrophilic” membrane, our results agree with their observations in the sense that $D_p$ is positive and increasing with increasing $\varepsilon _{2\text {m}}$. This is illustrated in Fig. 7. At the moment, we do not have data that generates a change in sign of $D_p$.

Like the Soret coefficient, the thermo-osmotic coefficient is related to the heat of transfer [3, 6, 7, 10] and presumably to $\Delta _\text {h,c} H$. As suggested by the linear relationship between $D_p$ and $\Delta H_\text {h,c}/\Delta _\text {h,c} T$ for different values of $m_2/m_1$ shown in Fig. 9, we tried the simple model,

22 $$\begin{aligned} D_{p,\text {model}} = a \frac{\Delta _\text {h,c} H}{\Delta _\text {h,c} T} + b \;. \end{aligned}$$

**Fig. 13:**
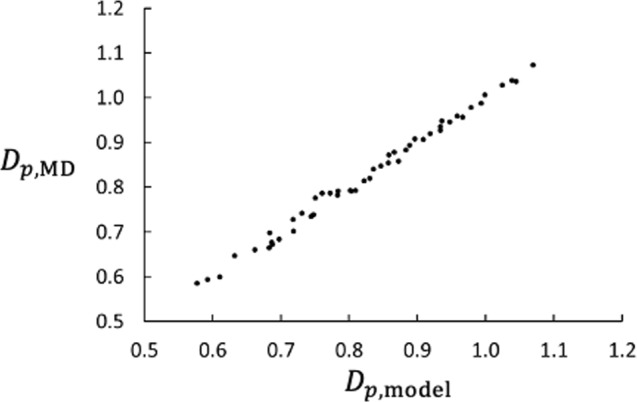
Check of NEMD results vs. model result for $D_p$. The model is given by Eq. (22). The data represent the 49 combinations of $m_2/m_1$ and $\varepsilon _{2\text {m}}$ used in this study

It turned out that this model with $a=1.057$ and $b=-3.47$ represents the MD data very well as shown in Fig. 13.

The Soret coefficient and the thermo-osmotic coefficient are related as given by the *L*-matrix, Eqs. (5)–(7). Equation (13) divided by Eq. (15) gives

23 $$\begin{aligned} \frac{S}{D_p} = \frac{L_{Dq}L_{VV}-L_{DV}L_{Vq}}{L_{Vq}L_{DD}-L_{VD}L_{Dq}} \end{aligned}$$

where the first term in the numerator refers to the heat of transfer due to $q_D^*$ and the second term refers to $q_V^*$. A study with extended set of conditions would give data for the individual *L*-coefficients that may substantiate this relation. What we do know, is that while the models in Eqs. (20) and (22) work for *S* and $D_p$, respectively, a model of the form Eq. (20) does not work for $D_p$ and Eq. (22) does not work for *S*.

We remind the reader that the membrane is extremely thin, a thermal insulator, and with nano-scale pore size, meaning that local thermal forces are very large (*cf.* Appendix B.3). Although this is not realistic for practical membranes, it has enabled a study of simultaneous thermodiffusion and thermo-osmosis in the same membrane. This work is a supplement to previous studies [13–19]. It gives a thermodynamic framework for the coupling between the two processes. There are different situations where such extreme conditions also apply, namely in shock waves. Earlier work has shown that component separation can occur over nanometer scale distances in a shock wave front where temperature gradients are as extreme as those used here [35].

We finally discuss the question we set out to help solve; how the knowledge gained can teach us how to construct membranes with large separation of components, or large pressure-buildups. From the knowledge of the enthalpy variation across the membrane we here learn that the larger the reduction in heavy component enthalpy across the membrane, the larger is the accumulation of the heavy component on the cold side, or the larger is the pressure buildup on this side. The fluid–membrane interactions that we are seeking preferably large.

An interesting question is whether and how the models for *S* and $D_p$ found here can be modified to other cases, such as different pore geometries and more realistic potential models. Clearly, the chemical nature of the membrane is important. Also neutral membranes can deliver separation work. Such membranes are probably less expensive than selective ones. Low-temperature waste heat may then become useful for separation work.

## Conclusion

The primary objective of this study was to find the effects of fluid–membrane interactions on thermodiffusion and thermo-osmosis, and the coupling between them. For a range of isotope mixtures and with varying interactions between mixture and membrane, we have found numerical support for two empirical models; one for the Soret coefficient $S = a_m (m_2-m_1)/(m_2+m_1) + b_{\varepsilon }(\varepsilon _{2m} -1)$, and one for the thermo-osmotic coefficient $D_{p} = a (\Delta _\text {h,c}H/\Delta _\text {h,c}T) + b$, where $a_m$, $b_\varepsilon$, *a*, and *b* are empirical parameters. The model for *S* is an extension of the common Chapman model [23]. The surprisingly simple models, in part adopted from bulk systems, have good potentials for working well for membrane systems. We suggest that the models for both *S* and $D_p$ be further tested.

We have found that a nanoporous membrane can have a significant impact on the Soret coefficient. If the properties of the fluid components are identical, except for adsorption preference for the membrane, there will still be a nonzero Soret coefficient caused by this preference. The membrane can strengthen/reduce the Soret coefficient if the lighter/heavier component adsorbs more strongly to the membrane. In other words, the separation efficiency increases if the lighter component is more strongly attracted to the membrane. This points to a role for enthalpy effects, which has indeed been found.

Remarkably, the empirical formulas for thermodiffusion and thermo-osmosis seem to be superimposed on one another; simple linear relationships can be added. The relationships can be used for simultaneous generation of separation and pressure differences, using temperature gradients as an only driving force. The design of membranes intended for separation of fluid components or for pressure accumulation may benefit from more insight in the underlying mechanisms for heat transfer. We hope that further analyses can add to the vision to make use of abundant resources of waste heat.

## Figures and Tables

**Fig. 14:**
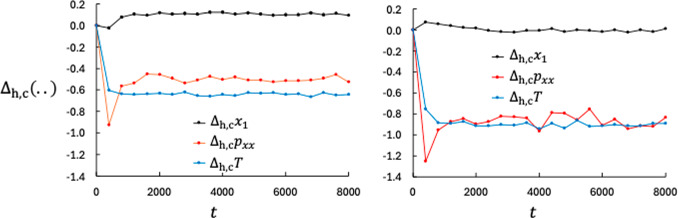
Three parameters used to check steady state in two different cases. The cases are $m_2/m_1=0.1$ (left) and $m_2/m_1=10$ (right). In both cases, $\varepsilon _{2\text {m}}=2.0$. The symbol $\Delta _\text {h,c}$ means difference between the cold and hot sides of the membrane

**Fig. 15:**
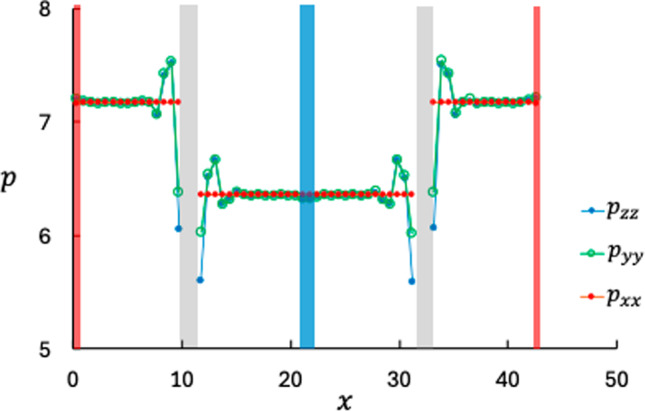
Three pressure components for the case with $m_2/m_1=10$ and $\varepsilon _{2\text {m}}=2.0$. The thermostated regons are marked red and blue and the position of the membranes are shown by the gray areas

**Fig. 16:**
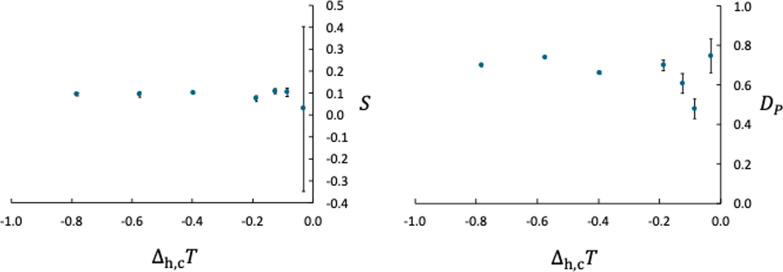
Computed Soret coefficient and thermo-osmotic coefficient as function of temperature difference across the membrane for the case with $m_2/m_1=10$ and $\varepsilon _{2\text {m}}=1.0$. The uncertainties were computed as described in Appendix B.4, method 1, in all cases

## Data Availability

Data may be made available from the corresponding author upon reasonable request.

## Appendix A Detailed derivation of Eqs. (5)–(10)

We start the derivation by considering the steady-state excess entropy production in the membrane, Eq. (3),

A1 $$\begin{aligned} \sigma ^\text {m} = J_u \Delta _\text {h,c} \left( \frac{1}{T} \right) - \sum _{k=1}^2 J_k \Delta _\text {h,c} \left( \frac{\mu _k}{T} \right) \end{aligned}$$

where $J_u$ is the energy flux, $J_k$ and $\mu _k$ are the mass flux and chemical potential of component *k*, respectively, and *T* is the temperature. The symbol $\Delta _\text {h,c}$ represents the difference “state c” - “state h.” Components 1 and 2 are the fluid components. The porous matrix is the third component and in Eq. (2), we have chosen it as a stationary frame of reference, i.e. $J_3 \equiv 0$, and therefore not included it explicitly in the entropy production.

To find working equations for thermo-osmosis and thermodiffusion, we expand $\mu _k/T$ as a function of temperature, pressure, and composition for an actual state around a reference state. Following Kjelstrup and Bedeaux[20], we choose this to be the membrane state “m”:

A2 $$\begin{aligned} \frac{\mu _k}{T}&= \left( \frac{\mu _k}{T} \right) ^\text {m} + H_k^\text {m} \Delta \left( \frac{1}{T} \right) + \left( \frac{V_k}{T} \right) ^\text {m} \Delta p \nonumber \\&\quad + \frac{1}{T^\text {m}} \Delta \mu _k^c + \text {higher order terms} \end{aligned}$$

where “$\Delta$” means the difference between the actual state and the reference state, *p* is pressure, and $\mu _k^c$ is the composition contribution to $\mu _{k}$. We have used

A3 $$\begin{aligned} \left( \frac{\partial (\mu _k/T)}{\partial (1/T)} \right) _{p,\{n_k\}} = H_k \end{aligned}$$

(the Gibbs–Helmholtz equation) and

A4 $$\begin{aligned} \left( \frac{\partial (\mu _k/T)}{\partial p} \right) _{T,\{n_k\}} = \frac{V_k}{T} \;. \end{aligned}$$

In Eq. (A4), $V_k$ is the partial molar volume of component *k*. From here on, we neglect higher order terms. Using Eq. (A2) in Eq. (A1), the linearized approximation is

A5 $$\begin{aligned}  &   \sigma ^\text {m} \approx J_q^{\prime \,\text {m}} \Delta _\text {h,c} \left( \frac{1}{T} \right) - \left[ \left( \frac{V_1}{T} \right) ^\text {m}J_1 + \left( \frac{V_2}{T} \right) ^\text {m}J_2 \right] \Delta _\text {h,c} p \nonumber \\  &   \quad - \left( J_1 \frac{\Delta _\text {h,c} \mu _1^c}{T^\text {m}} + J_2 \frac{\Delta _\text {h,c} \mu _2^c}{T^\text {m}} \right) \end{aligned}$$

Membrane properties (superscript “m”) are not known and hardly computable from data in the confinement of the pore. To proceed, we us the concept of Førland et al. [6], apply the expansion in Eq. (A2) to the actual states $( \mu _k/T )^\text {h}$ and $( \mu _k/T )^\text {c}$ and take the difference “c” - “h”. The properties with superscript “h” or “c” are extrapolated values of the actual bulk properties to the surface at the hot or cold side, respectively. The result is

A6 $$\begin{aligned} \Delta _\text {h,c} \left( \frac{\mu _k}{T} \right) = \overline{H_k} \Delta _\text {h,c} \left( \frac{1}{T} \right) + \overline{ \left( \frac{V_k}{T} \right) } \Delta _\text {h,c} p + \overline{\left( \frac{1}{T} \right) } \Delta _\text {h,c} \mu _k^c \end{aligned}$$

where $$\overline{H_k} = (H_k^\text {h}+H_k^\text {c})/2$$, $$\overline{V_k/T} = (V_k^\text {h}/T^\text {h}+V_k^\text {c}/T^\text {c})/2$$, and $$\overline{1/T} = (1/T^\text {h}+1/T^\text {c})/2$$.

In order to get an explicit dependence on the mole fractions $x_k$, we use $\mu _k/T^\text {m} = \mu _k^\text {m}/T^\text {m} + R \ln (\gamma _k x_k)$ where $\mu _k^\text {m}$ is the chemical potential of component *k* in the membrane state, *R* is the gas constant, and $\gamma _k$ is the activity coefficient of component *k* in the actual state. The difference across the membrane is

A7 $$\begin{aligned} \frac{\Delta _\text {h,c} \mu _k^c}{T^\text {m}}&= R \left[ \ln (\gamma _k^\text {c} x_k^\text {c}) - \ln (\gamma _k^\text {h} x_k^\text {h}) \right] \nonumber \\&= R \ln \left( \frac{\gamma _k^\text {c} x_k^\text {c}}{\gamma _k^\text {h} x_k^\text {h}} \right) = R \ln \left( \frac{x_k^\text {c}}{x_k^\text {h}} \right) \end{aligned}$$

Here, we have used that $\gamma _k^\text {c} = \gamma _k^\text {h}$ for the actual mixture used in this work. The logarithm in Eq. (A7) may be expanded around $x_k^\text {h}$ to give the linear approximation [3]

A8 $$\begin{aligned} \ln \left( \frac{x_k^\text {c}}{x_k^\text {h}} \right) \approx \frac{\Delta _\text {h,c} x_k}{x_k^\text {h}} - \frac{1}{2}\left( \frac{\Delta _\text {h,c} x_k}{x_k^\text {h}} \right) ^2 \end{aligned}$$

where $\Delta _\text {h,c} x_k= x_k^\text {c}-x_k^\text {h}$. Alternatively, it may be expanded around $x_k^\text {c}$:

A9 $$\begin{aligned} \ln \left( \frac{x_k^\text {c}}{x_k^\text {h}} \right) \approx -\ln \left( \frac{x_k^\text {h}}{x_k^\text {c}} \right) = \frac{\Delta _\text {h,c} x_k}{x_k^\text {c}} + \frac{1}{2}\left( \frac{\Delta _\text {h,c} x_k}{x_k^\text {c}} \right) ^2 \;. \end{aligned}$$

Taking the average of Eqs. (A8) and (A9) gives

A10 $$\begin{aligned} \frac{\Delta _\text {h,c} \mu _k^c}{T^\text {m}} \approx R \overline{\left( \frac{1}{x_k} \right) } \Delta _\text {h,c} x_k \end{aligned}$$

where $$\overline{(1/x_k)} = (1/x_k^\text {h}+1/x_k^\text {c})/2$$.

Finally, we use Eqs. (A6) and (A10) in Eq. (A2), which gives

A11 $$\begin{aligned}  &   \sigma ^\text {m} \approx \overline{J_q^\prime } \Delta _\text {h,c} \left( \frac{1}{T} \right) - \left[ \overline{\left( \frac{V_1}{T} \right) }J_1 + \overline{\left( \frac{V_2}{T} \right) }J_2 \right] \Delta _\text {h,c} p \nonumber \\  &   \quad - R \left[ J_1 \overline{\left( \frac{1}{x_1} \right) } - J_2 \overline{\left( \frac{1}{x_2} \right) } \right] \Delta _\text {h,c} x_1 . \end{aligned}$$

The average heat flux is $\overline{J_q^\prime } = (J_q^{\prime \, \text {h}}+J_q^{\prime \, \text {c}})/2$. By comparing Eqs. (A11) and (A5), we find working equations for the required membrane properties:

A12 $$\begin{aligned} J_q^{\prime \,\text {m}} \approx \;&\overline{J_q^\prime } \end{aligned}$$

A13 $$\begin{aligned} \left( \frac{V_k}{T} \right) ^\text {m} \approx \;&\overline{ \left( \frac{V_k}{T} \right) } \end{aligned}$$

The special case $J_k=0$ for all components gives $J_q^{\prime \,\text {m}} = J_u$, which is constant through the membrane at steady state.^3^

## Appendix B Computational details and uncertainty assessment

### B.1 Simulations

Prior to the production of NEMD runs, the initial configuration was generated with two membranes as illustrated in Fig. 1. Initially, both fluid and membrane particles were at FCC lattice positions. The membrane particles were fixed at these positions throughout the simulations. The MD box had periodic boundary conditions in all directions with 13,824 particles in total; 768 of which were membrane particles. The overall fluid composition was a 50/50 mixture. The two fluid components were uniformly distributed in the accessible parts of the MD box in the equilibrium start configuration.

The simulations were done with the in-house fortran code NEMD, which has previously been validated against LAMMPS [36] and used for simulations of several other transport processes. The fluid–fluid and fluid–membrane interactions were the Lennard–Jones/spline potential [21, 36]. The potential parameters are given in Tables 1 and 2. Each of the 49 cases were run $4\times 10^6$ time steps, each step was $\delta t = 0.002$ in Lennard–Jones units, i.e. a total length of 8,000 time units.

Given the narrow pores, we expected that the transient period from simulation start until steady-state data could be used for data analysis would be very long. The evolution of all the runs was monitored. Two examples of the evolutions of three key parameters, $\Delta _\text {h,c} T$, $\Delta _\text {h,c} p_{xx}$, and $\Delta _\text {h,c} x_1$ are shown in Fig. 14. The symbol $\Delta _\text {h,c}$ means difference between the cold and hot sides of the membrane. The cases were chosen as expected worst cases with strong adsorption of component 2 in the membrane, with $\varepsilon _{2\text {m}}=2.0$.

The transient period is most clearly shown in the pressure response. When the thermostats are switched on and the fluid in the bulk regions become hot and cold, there is a brief pressure surge until the steady state is established. Based on these data, we decided to use the last $2\times 10^6$ time steps (after $t=4000$) as steady-state data for computing averages.

### B.2 Calculation of pressure, heat flux, and extrapolation of bulk properties to the membrane surface

The pressure in the bulk regions was calculated as described by Ikeshoji et al. [37]. Typical results are shown in Fig. 15 for a case with $m_2/m_1=10$ and $\varepsilon _{2\text {m}}=2.0$. The membrane acts as a surface or wall with density oscillations due to packing effects and pressure oscillations in the directions parallel to the membrane. These oscillations extend 2–3 molecular diameters into the bulk. The pressure normal to the membrane, $p_{xx}$, is constant at steady state in accordance with the mechanical balance in the stagnant situation. The pressure was not computed in the pores. The reported pressure differences across the membrane were obtained from extrapolations of $p_{xx}$ from the bulk to the membrane surface.

**Table 3:** Test of local equilibrium in the bulk fluid for the case with $m_2/m_1=10$ and $\varepsilon _{2\text {m}}=1.0$

Method	*T*	$\nabla T$	$\rho$	$\nabla \rho$	*p*	$p^\text {excess}$
NEMD simulation	3.64	-0.06	0.649	0.003	6.67	4.31
Equilibrium simulation	3.61	0	0.649	0	6.61	4.27
Equation of state	3.64	N/A	0.649	N/A	6.57	4.21

The columns $\nabla T$ and $\nabla \rho$ show the gradients in temperature and density, respectively, in the hot bulk fluids in the NEMD simulations. The uncertainties in the NEMD numbers are discussed in Appendix B.4

The heat flux in *x*-direction, which was used in Eqs. (16) and (17), was computed in each layer (CV) as given by Evans and Morriss [38]:

B14 $$\begin{aligned} J_q^\prime= &   \frac{1}{V_{\text {CV}}}\sum _{i\in \text {CV}}\left[ \left( \frac{1}{2}m_i ( \textbf{v}_{i}-\textbf{v} )^{2}+\phi _{i}\right) \left( v_{i,x}-v\right) \right. \nonumber \\  &   \left. -\frac{1}{2} \sum _{\begin{array}{c} j=1 \\ j \ne i \end{array}}^{N}\left[ \left( \textbf{v}_{i}-\textbf{v}\right) \boldsymbol{\cdot } \textbf{f}_{ij}\right] x_{ij}\right] \end{aligned}$$

where $V_\text {CV}$ is the layer volume, $\textbf{v}_i$ is the velocity of particle *i*, $\phi _{i}$ is the potential energy of particle *i* in the field of all the other particles within range (including those outside the $\text {CV}$), $\textbf{f}_{ij}$ is the force acting on *i* due to *j*, and $x_{ij}=x_j - x_i$ is the distance from *i* to *j* in *x*-direction. At steady state, the flow velocity $\textbf{v}$ and its *x*-component *v* are zero and the heat flux is constant through the bulk phases.

Other properties such as temperature and mole fraction were found by extrapolation after fitting linear functions to NEMD data in the bulk regions (see Fig. 4). The MD box had two membranes as shown in Fig. 1 and mirror symmetry around $x=L_x/2$. Data from the two halves were pooled to use all the available information and assess uncertainties in the results (see Appendix B.4).

### B.3 Local equilibrium and effect of thermal force strength

The large thermal force used in the simulations (approximately 100 K across the membrane with thickness approximately 1 nm) raises the concern that the system may not be in local equilibrium. We examined this issue in three ways, using the case with $m_2/m_1=10$ and $\varepsilon _{2\text {m}}=1.0$ as example: (1) the temperature gradient was varied and the effects on *S* and $D_p$ were checked, (2) by comparing selected thermodynamic properties in the non-equilibrium bulk fluid with the value obtained from equilibrium simulations, and (3) comparing the same properties with results obtained from the equation of state [39].

The results of test 1 are shown in Fig. 16. We did not observe a clear trend in *S* and $D_p$ as a function of $\Delta _\text {h,c} T$ and we concluded that the large temperature differences used in the production runs were in accordance with the assumption of local equilibrium. The uncertainties given in Fig. 16 were estimated as described in Appendix B.4, method 1.

A set of *T* and $\rho$ in the bulk fluid was used for tests 2 and 3. The set was used as input to an equilibrium simulation for the same bulk fluid in a MD box without membranes and in the Thermopack package (https://www.sintef.no/en/software/thermopack/) based on the equation of state developed by Van Westen et al. [39]. The resulting bulk pressures are given in Table 3. The equilibrium simulation was a basic NVE simulation with $2 \times 10^6$ steps after equilibration. Since the resulting temperature did not exactly match the input temperature, we have included both the total pressure *p* and the excess pressure $p^\text {excess} = p - p^\text {ideal gas}$ in the table. The agreement is good with the EoS pressure slightly below the simulation pressures.

### B.4 Uncertainty assessment

Errors in the computed averages were estimated in three different ways: 1. The symmetry of the MD box was used to compute two equivalent differences across the left and right membranes. The reported results were the averages of the pair and the error was computed as $| \Delta _\text {h,c}(\text {left})-\Delta _\text {h,c}(\text {right})| /2$ where “left” and “right” represent values of a property across the the left and right membrane, respectively.

2. The steady-state period was used to collect sub-averages. These sub-averages were then used to compute grand averages for the entire steady-state period and random errors.

3. For the case with $m_2/m_1=10$ and $\varepsilon _{2\text {m}}=1.0$, we did six independent runs starting from different configurations. Each of the starting configurations originated from the same FCC lattice and went through different numbers of Monte-Carlo steps. The six final MC configurations were then used as starting configurations for six NEMD runs. Each of these were then analyzed in the same way as all the others, and the grand average and uncertainties were determined from the variance in the results.

On average, the errors were found to be approximately 0.01 in both *S* and $D_p$.

## Footnotes

1 The uncertainties in the results are equal to or smaller than the size of the symbols used in the figures unless otherwise indicated with error bars. See also Appendix B.4.

2 Haugen and Firoozabadi used the term “pressure diffusion” for the contribution to the *diffusive* mass flux driven by a pressure gradient [32]. In principle, this contribution is present also in bulk fluids, but it is small and usually neglected. In packed thermogravitational columns and geological formations, gravity gives a hydrostatic pressure gradient, which is an external body force. This must not be confused with thermo-osmosic pressure, which is the system’s response to a temperature gradient.
